# Translation and cross-cultural adaptation of a questionnaire for assessing hyperacusis in Williams syndrome

**DOI:** 10.1055/s-0045-1811624

**Published:** 2025-09-08

**Authors:** Jacqueline Aquino do Nascimento, Lucas Pinto Mielle, Liliane Aparecida Fagundes Silva, Alessandra Giannella Samelli, Carla Gentile Matas

**Affiliations:** 1 Universidade de São Paulo, Faculdade de Medicina, Departamento de Fisioterapia, Fonoaudiologia e Terapia Ocupacional, São Paulo SP, Brazil.

**Keywords:** Williams Syndrome, Hyperacusis, Audiology, Translating, Surveys and Questionnaires

## Abstract

**Background:**

Williams syndrome (WS) is a genetic, multisystemic, and neurodevelopmental disorder. The prevalence of auditory hypersensitivity in WS is high, especially in childhood, with reports in the literature from 94 to 100% of individuals evaluated, which can generate significant impacts on their quality of life. Therefore, the existence of instruments for screening hyperacusis that are easy and quick to apply for use in clinical routine is essential.

**Objective:**

To translate and perform the cross-cultural adaptation of the Hyperacusis Screening Questionnaire into Brazilian Portuguese.

**Methods:**

A questionnaire concerning auditory hypersensitivity in WS was translated by two translators who are fluent in English. After the synthesis of the translations, back-translation was performed to analyze similarity, a meeting of the expert committee for semantic and linguistic adaptation of the instrument was held, and pretesting and validation of content and appearance was conducted.

**Results:**

The translated and adapted version of the questionnaire was similar to the original regarding general and referential meaning. Answers from 324 families of individuals with WS were included, 85.2% of those reported hyperacusis as a ongoing symptom.

**Conclusion:**

The translation and cross-cultural adaptation of the questionnaire were performed according to the methodology recommended in the literature, with necessary equivalences being made for the Brazilian reality. The instrument developed and tested in the present study proved to be useful in screening for hyperacusis in the population with WS, allowing its use in future investigations on the subject and comparison with other studies.

## INTRODUCTION

Williams syndrome (WS) is a genetic, multisystemic, and neurodevelopmental disorder that occurs in between 1:7,500 and 1:20,000 live births. 1 2 It is caused by a microdeletion in the chromosomal region 7q11.23, which affects ∼ 28 genes, and is equally prevalent among men and women, occurring in all types of populations around the world. 3

Heart problems, such as supravalvular aortic stenosis, changes in calcium metabolism, characterized by infantile hypercalcemia, as well as connective tissue abnormalities, are some of the afflictions that make up this condition. 1

Hearing abnormalities are also among the most frequent findings, including progressive sensorineural hearing loss and high prevalence of conductive impairments. Even in individuals with normal hearing sensitivity, subclinical signs of hearing dysfunction can be detected, as evidenced by reduced otoacoustic emission amplitudes. 4 5 6 7

According to the current state of the medical literature, such alterations occur mainly due to the absence of some genes that are important for the maintenance of the auditory system’s homeostasis, including *ELN* , *LIMK1* and *GTF2IRD1.* 8 9 10 11 Furthermore, changes are also observed in the perceptual processing of acoustic signals, as evidenced by auditory hypersensitivity. This can manifest in various forms: fascination, marked by an abnormal attraction to sound stimuli; phonophobia, characterized by an exaggerated fear or alert response to specific sounds; and hyperacusis, defined as an increased sensitivity to sounds of low to moderate intensity, which may result in discomfort or pain. 12 13 14 15

The incidence of auditory hypersensitivity in WS is high, especially in the pediatric population. 15 The modern literature shows that complaints of hypersensitivity can be observed in 94 to 100% of the evaluated individuals, 16 17 18 and it can be moderate to severe in 84% of individuals with WS. 19

Several hypotheses have been proposed regarding the causes of hyperacusis in WS. Some studies suggested that hyperacusis could be related to distortions in the neural encoding of auditory input, leading to an abnormal perception of loudness, or even to a failure of the central nervous system to habituate to auditory stimuli. 16 17 Furthermore, other studies have linked hyperacusis to a possible loss of outer hair cell inhibition caused by lack of modulation of the medial olivocochlear efferent system, 19 serotonin dysfunction, 20 reduced expression of the *LIMK1* gene, 21 and a combination of cochlear hearing loss and auditory nerve dysfunction, which would probably alter the perception of loudness. 9 Despite this, the pathophysiological mechanisms are still not fully understood. 9 19 22

The present study is justified by the fact that auditory hypersensitivity is a persistent condition that significantly impacts the quality of life of affected individuals. Therefore, the availability of validated screening instruments that are both quick and easy to administer in clinical settings is essential to facilitate appropriate treatment.

Therefore, the objective of the present study was to translate, cross-culturally adapt, and validate a questionnaire to assess the clinical characteristics of hyperacusis in subjects with WS, and to measure the impact of this symptom on daily activities.

## METHODS

The present study was approved by the research ethics committee of the institution where it was developed, under the number 2,504,522. The use of the Hyperacusis Screening Questionnaire 19 was authorized by its author.

### Instrument description

The Hyperacusis Screening Questionnaire was developed by Gothelf et al. 19 with the objective of studying the clinical characteristics of hyperacusis presented by individuals with WS. The questionnaire consists of five questions, the first two of which are: “Is your child presently frightened or bothered by certain sounds?” and “If your child is not currently bothered by certain sounds, was this a past problem?” If at least one of the two questions was answered positively, the families were asked to complete the questionnaire. Question 3 is an open question regarding the age at which the problem was most evident. Question 4 is a multiple-choice question (5 options), in which the caregiver is asked to indicate how much the sensitivity to sounds interferes with the individual's daily life activities, ranging from “No interference” to “Very severe interference”: Finally, in question 5, a series of familiar sounds are presented and the caregiver is asked to indicate whether the individual with WS is currently bothered by the sound, whether they have been bothered in the past and what is the individual's typical response to that sound ( **Supplementary Material II** – available at https://www.arquivosdeneuropsiquiatria.org/wp-content/uploads/2025/07/ANP-2025.0101-Supplementary-Material-II.docx ).

### Procedures

The present study was conducted between November 2023 and July 2024. The questionnaire was translated and cross-culturally adapted, following the steps below:

- Translation and synthesis of the translations: In this stage, two independent translations were performed by two different professionals who are fluent in English, generating versions T1 and T2, and a synthesis of the two translations was performed by two audiologists, who made the adaptations and reformulations they deemed necessary, resulting in the synthesis version of the initial translations, which was called version T12.
- Back-translation: To analyze the similarity of meaning and content between the summary version and the original version, the back-translation was performed by two other independent and bilingual translators who had no knowledge of the original version. Each translator produced a new version of the instrument, called RT1 and RT2, which proved to be compatible with the original version of the questionnaire, demonstrating the accuracy of the translation. In the end, a synthesis of these two back-translations was created (RT12).
- Experts committee: The aim of this meeting was to analyze the semantic, idiomatic, experiential, and linguistic equivalences of the translated version in comparison with the original version of the instrument, to produce the preliminary final version of the questionnaire ( **Supplementary Material III** – available at https://www.arquivosdeneuropsiquiatria.org/wp-content/uploads/2025/07/ANP-2025.0101-Supplementary-Material-III.docx ).
- Pretest: The final version of the questionnaire was edited electronically using the Google Forms platform and was sent by the Brazilian Williams Syndrome Association to families of patients with WS to verify how easy it was to understand the questionnaire according to its content and form.
- Final stage: After the questionnaire responses were returned, the committee of experts held a consensus meeting on the version applied to the participants to check for possible adjustments that needed to be made.

### Data analysis

Given the high variability in responses related to both chronological age and the age at which hyperacusis was most bothersome (question 3), it was decided to categorize the data according to life stages for better summarization: early childhood (0–6 years old), middle childhood (7–12 years old), adolescence (13–17 years old), and adulthood (> 17 years old). Furthermore, some caregivers indicated as “always”, and others reported an age group that included more than one categories; this variation was taken into account in the analysis.

For question 4, the responses were transformed into scores between 0 and 4, as follows: 0 - No interference; 1 - Mild interference; 2 - Moderate interference; 3 - Severe interference; 4 - Very severe interference.

The results were compared by one-way analysis of variance (ANOVA), according to the age group of the individual with WS at the time of answering the questionnaire (early childhood, middle childhood, adolescence, or adulthood).

To analyze the reaction to sounds (question 5), the percentage of responses that stated that they felt discomfort with each specified sound, both currently and in the past, was calculated. In addition, the reactions most frequently described by caregivers were collected to summarize the main reactions that individuals with WS presented to sounds.

## RESULTS

### Pretest

In total, 324 families of individuals with WS responded to the survey. The patients had a mean age of 15.57 years old and there was no significant difference between the sexes ( *p* = 0.617). Table 1 shows the demographic characteristics of the sample and the distribution of complaints related to hyperacusis (related to questions 1 and 2 of the questionnaire).

**Table 1:** Sample characterization

	Min	Max	Mean	SD
**Age (years old)**	1	67	15.57	10.13
		**N**		**%**	
**Sex**	Male	167		51.5	
	Female	157		48.5	
**Complaints**	Current complaint of hyperacusis	276		85.2	
	Past complaint of hyperacusis	36		11.1	
	No history of hyperacusis complaints	12		3.7	

Abbreviations: Min, minimum; Max, Maximum; N, number of individuals; SD, standard deviation.

A high prevalence of hyperacusis can be observed in the population with WS, as this complaint was verified in 85.2% of the individuals who completed the questionnaire. In addition, 11.1% of the cases reported having experienced this complaint at some point in their lives, but that it was no longer a current complaint, and only 3.7% of the cases reported never having experienced hyperacusis.

**Figure 1:**
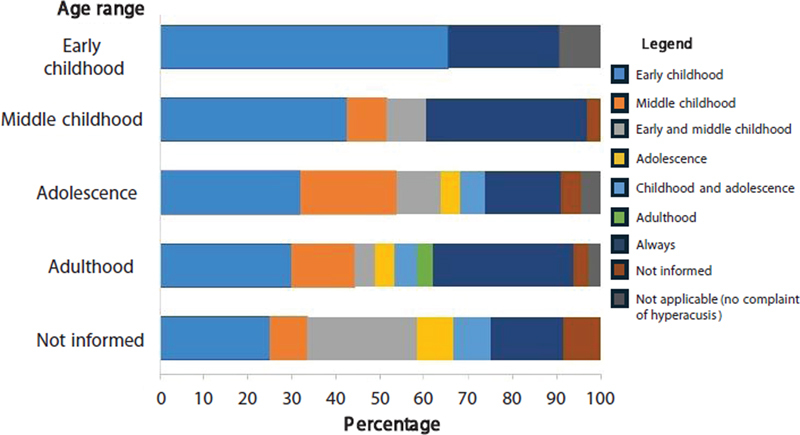
Stage of life in which individuals reported the worst impact of hyperacusis, classified according to chronological age range at the time of questionnaire answer (y-axis).

Regarding question 3, it can be observed that the families reported a greater impact of hyperacusis during early and middle childhood ( Figure 1 ).

In question 4, regarding how much sensitivity to sounds interferes with daily functions, the score obtained was between 0 and 4, with an average of 2.21 points (moderate interference) and standard deviation (SD) of 1.15, and there was no difference according to the patient's age group classification ( *F* (4) = 0.450; *p* = 0.772).

As for question 5, the sounds most frequently reported as causing discomfort were fireworks, machines/electric tools, thunder, shouting, and balloons popping ( Table 2 ).

**Table 2:** Percentage of individuals who reported discomfort for each sound (question 5), considering complaints that are still present or that occurred at some point in the past ( *n* = 324)

	Present		Past	
	N	%	N	%
Electric machine	183	56.5	77	23.8
Firework	201	62.0	69	21.3
Thunder	182	56.2	59	18.2
Balloon popping	175	54.0	72	22.2
Shouting	177	54.6	66	20.4
Loud conversation	102	31.5	80	24.7
Loud music	77	23.8	88	27.2
Siren/alarm	149	46.0	80	24.7
Motor vehicle	138	42.6	82	25.3
Barking	107	33.0	82	25.3
Whistle	121	37.3	76	23.5
Hammer	70	21.6	88	27.2
Telephone	14	4.3	85	26.2
TV	21	6.5	81	25.0

In Table 3 , you can see the percentage of the main reactions described when the patients were exposed to different sounds that cause discomfort.

**Table 3:** Percentage of cases that reported presenting each of the reactions ( *n* = 324)

	N	%
Covers ears	158	48.8
Becomes irritated	147	45.4
Reacts by crying	97	29.9
Becomes scared	73	22.5
Becomes anxious	61	18.8
Reports pain	12	3.7
Sweats	6	1.9

### Adaptation of the questionnaire

Regarding the cross-cultural adaptation of the questionnaire, the Portuguese translation resulted in a version that was easily understood by Brazilian Portuguese speakers, requiring only minor modifications. These adjustments primarily involved the inclusion of terms more commonly used in the Brazilian context and grammatical refinements. The original versions, a summary of the translations, the back-translation summaries, and the version reviewed by the expert committee are presented in Table 1 .

During the synthesis stage of the T1 and T2 translations, the term “celular” was included alongside “telefone,” since it’s most commonly used in the Brazilian context. No additional linguistic or cultural modifications were considered necessary.

Subsequently, the back-translation was performed. At this stage, a great similarity was observed between the back-translated versions and the original questionnaire, and the differences found were detailed in **Supplementary Material I** – available at https://www.arquivosdeneuropsiquiatria.org/wp-content/uploads/2025/07/ANP-2025.0101-Supplementary-Material-I.docx .

During the pretest stage, all participants reported that the questionnaire was easy to understand in terms of its content and format. As a result, no further modifications were necessary for the final version, supporting the validity and applicability of the questionnaire for use with the Brazilian population.

## DISCUSSION

In the present study, a specific questionnaire for hyperacusis in WS was translated and cross-culturally adapted. The translated version was then validated in a sample of 324 families of individuals with WS, most of whom were children and adolescents, with no significant difference between the sexes.

Regarding hyperacusis, 96.3% of individuals reported experiencing this complaint at some point in their lives, with 85.2% indicating that it was a persistent issue. Among adult patients, it was observed that in 58.38% of cases, hyperacusis was most prominent between early childhood and adolescence.

Despite the higher incidence among the younger population, it can be observed that, on average, hyperacusis caused moderate interference—defined as sounds significantly distracting the individual or interrupting their activities—highlighting the impact of this symptom on the daily lives of patients with WS, regardless of their age group.

Nigam et al. 17 described that this alteration could be observed in ∼ 95% of individuals with WS. In a study conducted in 2005 by Levitin et al., 13 it was observed that of the 118 individuals in the study group, 79.8% presented with complaints of phonophobia and 90.6% had a reduced pain threshold when compared with individuals with typical development.

Similarly, Gothelf et al. 19 evaluated a group of 49 children with WS through questionnaire and electrophysiological examinations, observing that 84% of these children presented moderate to severe hyperacusis, which was more pronounced in childhood and tended to improve during adolescence. In addition, lower discomfort thresholds were noted compared with individuals with typical development.

Regarding the sounds that cause the greatest discomfort, the most prominent are fireworks, machines/electric tools, thunder, shouting, and balloons popping. These results corroborate those reported by Gothelf et al. 19 using the original questionnaire, in which the authors observed complaint rates of 62.8% for fireworks, 67.3% for machine/power tools, and 62.8% for thunder. The reason for the high occurrence of hyperacusis in WS is still discussed; however, there is evidence of the interference of the deletion of specifics genes in this process. One of the genes associated with these alterations is *LIMk1* , which can increase the amplitude of the electromobility of outer hair cells and decrease its extension, thus impairing the modulation of cochlear amplification, possibly leading to hyperacusis. 9 Johnson et al. 20 investigated the possible relationship between the efferent auditory system and hyperacusis in WS. To this end, they assessed a group of nine individuals with WS using pure tone audiometry, immittance testing, and transient otoacoustic emissions (TEOAE), and found that three individuals had sensorineural hearing loss and no TEOAE, and four individuals had normal hearing thresholds with no TEOAE. The authors highlighted the cochlear involvement in this alteration and supported the hypothesis that the origin of hyperacusis is linked to efferent modulations of outer hair cells.

Another gene that could be associated is *GTF2IRD1* , which is linked to craniofacial and neurological abnormalities in WS. In 2015, Canales et al. 11 investigated the relationship between the deletion of this gene and hyperacusis in guinea pigs and observed reactions consistent with hyperacusis in this group that had the deletion of this gene, with significant results when compared with the group without this alteration.

Additionally, the influence of the ELN gene – which is responsible for elastin synthesis, particularly in blood vessels – was examined. A reduction in its expression may impair cerebral blood vessels, potentially leading to damage and dysfunction of the central nervous system. Furthermore, its action on the tip links of cochlear hair cells could lead to an asynchrony of stereocilia and neuronal activation. 19

Although the pathophysiology of hyperacusis in WS remains unclear, this auditory disorder is known to cause fear, discomfort, anxiety, and pain, ultimately reducing the quality of life of those affected. 23 24 In children, hyperacusis can interfere with daily activities, hinder academic performance, and negatively impact social interactions.

In this context, the use of validated questionnaires enables effective screening and provides a basis for counseling caregivers to help them avoid sounds that may cause discomfort or trigger negative emotions such as irritation, fear, or anxiety. Moreover, identifying the complaint allows for targeted interventions aimed at desensitizing hyperacusis, while the continued use of validated questionnaires supports the monitoring of progress following intervention.

Another important aspect is the high prevalence of hearing loss among individuals with WS. This underscores the need for regular audiological monitoring, particularly in those with hyperacusis, as the presence of hyperacusis may signal underlying cochlear damage.

The questionnaire that was translated and adapted in the present study is quick and easy to administer and can be self-reported by caregivers either at home or in a waiting room, without affecting consultation time. Additionally, it does not require specialized equipment for its use – unlike, for example, the discomfort threshold test, which demands specific environments, trained professionals, and technical devices, as well as the patient's sufficient cognitive ability to respond reliably to the assessment.

By using the translated and adapted version of the Hyperacusis Screening Questionnaire, it was possible to observe rates of hyperacusis in individuals with WS that closely align with those reported in the international literature. This suggests that the instrument is a valuable tool for screening hyperacusis complaints in the WS population, potentially facilitating timely and appropriate treatment. Additionally, it provides an opportunity for further research in the field and contributes to a deeper understanding of how this condition affects the daily quality of life in this population.

As the questionnaire was administered remotely, without the presence of a professional to mediate its application, some responses contained missing information or lacked standardization across families. This represents a limitation of the present study, as it hindered the possibility of conducting more robust analyses.
